# Nanogroove-Enhanced Hydrogel Scaffolds for 3D Neuronal Cell Culture: An Easy Access Brain-on-Chip Model

**DOI:** 10.3390/mi10100638

**Published:** 2019-09-23

**Authors:** Alex Bastiaens, Sijia Xie, Regina Luttge

**Affiliations:** 1 Neuro-Nanoscale Engineering Group, Department of Mechanical Engineering and Institute of Complex Molecular Systems (ICMS), Eindhoven University of Technology, 5600 MB Eindhoven, The Netherlands; a.j.bastiaens@tue.nl; 2 MESA+ Institute for Nanotechnology, University of Twente, 7500AE Enschede, The Netherlands; sijiaxie1987@gmail.com

**Keywords:** 3D cell culture, neuronal cells, SH-SY5Y cells, image-based screening, nanogrooves, neuronal cell networks, neuronal guidance

## Abstract

In order to better understand the brain and brain diseases, in vitro human brain models need to include not only a chemically and physically relevant microenvironment, but also structural network complexity. This complexity reflects the hierarchical architecture in brain tissue. Here, a method has been developed that adds complexity to a 3D cell culture by means of nanogrooved substrates. SH-SY5Y cells were grown on these nanogrooved substrates and covered with Matrigel, a hydrogel. To quantitatively analyze network behavior in 2D neuronal cell cultures, we previously developed an automated image-based screening method. We first investigated if this method was applicable to 3D primary rat brain cortical (CTX) cell cultures. Since the method was successfully applied to these pilot data, a proof of principle in a reductionist human brain cell model was attempted, using the SH-SY5Y cell line. The results showed that these cells also create an aligned network in the 3D microenvironment by maintaining a certain degree of guidance by the nanogrooved topography in the z-direction. These results indicate that nanogrooves enhance the structural complexity of 3D neuronal cell cultures for both CTX and human SH-SY5Y cultures, providing a basis for further development of an easy access brain-on-chip model.

## 1. Introduction

Current models to study the brain and brain diseases are limited in their capabilities to translate findings toward the discovery of drugs that help treat these diseases [1]. In particular, the failure rate in drug development for brain diseases is disproportionately high compared to other drug discovery areas [2] and has yet to provide drugs that can slow, halt or reverse neurodegenerative diseases such as Alzheimer’s disease (AD) [3] or Parkinson’s disease (PD) [4]. While improvements can be made toward animal model studies, another approach is to study in vitro models. The so-called organ-on-chips (OOC) technology provides an opportunity to study human cells or organoids in a physiologically relevant microenvironment, potentially bridging the gap between current pre-clinical studies and human-based clinical trials [5,6].

To study the brain and brain diseases in an OOC platform, coined a brain-on-chip (BOC), we require a well-designed microsystem that can incorporate an environment for brain cells in a culture which mimics structural complexity in 3D [7]. In the natural cerebral cortex, a layered construction is formed during development. Through the cortical layers, so called “cortical columns” consist of aggregated cell bodies and neuronal outgrowths that are alternatively organized in laminar layers, and perpendicularly distributed in each layer as columns [8,9]. It is reported that neurons locating at the same radial column show similar response properties recorded by microelectrodes [10]. While brain organoids can be cultured and exhibit such 3D structural complexity [11], there is little control over the location of regions, where specific brain cell types or structures can be generated or the actual control of the distal arrangement of cells to each other. The use of micro- and nanotechnology can aid the design of BOC platforms that offer more control of these parameters and potentially more reproducible experiments.

Current research shows that nanotopography can guide neuronal cells’ outgrowth and hence neuronal cell network organization, thereby creating more in vivo-like structures in in vitro models [12,13,14,15,16,17,18,19]. Previously, our group has investigated a range of nanogrooved patterns in different substrate materials and their impact on primary rat brain cortical (CTX) cells and the neuroblastoma cell line SH-SY5Y in 2D cultures. We have shown, in these studies, that slight dimensional changes in these nanogroove patterns elicit different responses with regards to the extent of the guidance effect, or alignment, for both CTX cultures and SH-SY5Y neuronal outgrowths [20,21]. Specifically, a pattern with a ridge width of 230 nm and pattern periodicity of 600 nm provided good alignment results for CTX cultures, as compared to other nanogroove dimensions. Higher alignment was also seen for SH-SY5Y cells when cultured on patterns with a ridge width of 230 nm and pattern periodicity of 1000 nm, compared to other nanogroove dimensions. Based on an automated image-based screening method we developed, it was shown that for SH-SY5Y cells, a smaller ridge width compared to the pattern periodicity of a nanogrooved pattern resulted in an increased alignment of neuronal outgrowths. Also, an increase in alignment of such outgrowths correlated to increased differentiation of SH-SY5Y cells and increased outgrowth length [22]. The material properties of the nanogrooved substrate material, here the stiffness of either silicon, glass or polydimethylsiloxane (PDMS), has shown to influence neurite length, too [23]. We first investigated if our previously developed automated imaged-based neuronal network screening method also applied to 3D CTX cell cultures. Since the method was successfully adopted in the data set of the pilot study, a proof of principle in a reductionist human brain cell model was attempted using also the SH-SY5Y cell line in 3D. In this paper, it is our aim to demonstrate that it is possible to control outgrowth direction in 3D by simply applying soft scaffolds, such as Matrigel, atop of the cells initially seeded on nanogrooved substrates. The benefits of a 3D microenvironment using a hydrogel, and the interfacing with a nanogrooved substrate, are complementary, and enable us to generate structural complexity in BOC platforms.

Applying this new culture concept, the cells will form a cellular network on top of the nanogrooves and inside the 3D volume of the hydrogel. By performing immunofluorescence staining and subsequent confocal microscopy, the cell cultures can be visualized. However, to analyze the generated data quantitatively, our previously developed automated image-based screening method was used to assess outgrowth alignment and length throughout the 3D volume of the cell cultures. In this paper, we describe how the screening method was tested using z-stack data of astrocytes in a 3D CTX culture on nanogrooves and subsequently applied to a human brain cell model culture experiment using SH-SY5Y. This type of data analysis confirmed that these neuronal cell cultures both contain a degree of outgrowth alignment, both at the nanogrooved substrate surface, as well as up to several micrometers away from that surface.

The results further show that the concept of nanogrooved-enhanced 3D scaffolds provides structural complexity in a reductionist human brain model. This type of BOC culture format hence offers a viable and accessible tool for creating more in vivo-like brain models using nanotechnology, thereby bringing advances in our understanding of the brain and brain disease.

## 2. Materials and Methods

### 2.1. Nanogroove Substrate Fabrication

**Figure 1:**
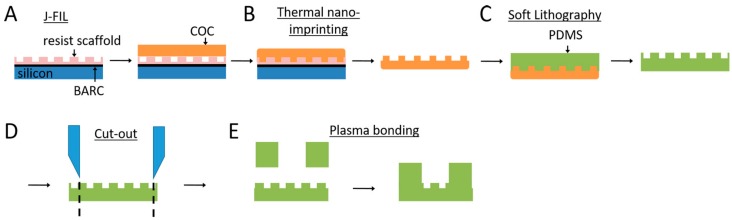
Fabrication process of polydimethylsiloxane (PDMS) constructs for 3D neuronal cell culture on nanogrooved substrates. (**A**) Nanoresist patterning through jet and flash imprint lithography on a silicon wafer coated with a bottom anti-reflective coating using a quartz master. (**B**) Thermal nano-imprinting of the nanogrooved patterns in the nanoresist to a negative mold in cyclic olefin copolymer. (**C**) Soft lithography from the negative mold into a layer of polydimethylsiloxane (PDMS). (**D**) A 10 mm puncher was used to cut out the required nanogrooved pattern from the PDMS. (**E**) A PDMS ring was cut out using punchers. After plasma oxidation of both the nanogrooved PDMS from (**D**) and the PDMS ring, these parts were bonded to create a PDMS construct with a well for 3D neuronal cell culture containing a nanogrooved substrate on the bottom.

The fabrication details for the nanoresist scaffolds were previously published by Xie and Luttge [20]. In brief, nanoresist was patterned using jet and flash imprint lithography (J-FIL) on a standard double-sided polished 100 mm diameter silicon wafer (Figure 1A). The wafer was first coated with a bottom anti-reflective coating (BARC; DUV30J, Brewer Science, Rolla, MO, USA) layer using a quartz master, kindly provided by the Bijkerk group at the University of Twente. The nanogrooved patterns had dimensions in the range of 200–2000 nm pattern periodicity, a ridge width of 100–1340 nm, and a height of 118 nm. Subsequently, the nanoresist patterns were used directly as a template in thermal nanoimprint lithography, creating a negative copy in cyclic olefin copolymer (COC; optical grade TOPAS 8007S-04, Topas Advanced Polymers, Frankfurt am Main, Germany) using a thermal nanoimprint lithography system (EITRE 6, Obducat, Lund, Sweden) at 108 °C and applying a pressure of 4 MPa (Figure 1B). After cooling the COC to room temperature, it was peeled off the nanoresist and was ready to be used as a negative mold for further replication steps.

Replication into culture substrates was performed using soft lithography of PDMS (Sylgard 184, Dow Corning, Midland, MI, USA) on either the nanoresist or the COC molds (Figure 1C). As the original nanoresist master contained complementary nanogrooves dimensions, e.g., complementary ridge widths of 230 nm and 370 nm with a 600 nm pattern periodicity, PDMS copies with similar dimensions could be obtained from both the nanoresist and the COC molds. PDMS elastomer and curing agent were mixed at a 10:1 ratio and degassed for 10 min using a vacuum desiccator. PDMS was spin-coated onto the mold at 500 rpm for 60 s to achieve a 100 µm layer of PDMS. Subsequently, the PDMS was cured in an oven at 65 °C for 4 h and peeled off for the nanogrooved PDMS cell culture substrates in these experiments.

For the SH-SY5Y cells, the nanogrooved patterns with a 1000 nm pattern periodicity and 230 nm ridge width, copied from the COC mold, were used in the experiments. A 10 mm diameter punch was used to punch out this specific nanogrooved pattern from the range of patterns (Figure 1D). A 100 µm layer of PDMS was spin-coated onto a 100 mm diameter silicon wafer and cured in the same way as described for the COC mold. The layer of PDMS was peeled off and placed in a polystyrene Petri dish. A 10 mm and a 3 mm punch were used to punch out rings of the same size as the nanogrooved PDMS, with a hole in the center. Plasma oxidation (EMITECH K1050X, Quorum, Lewes, UK) at 50 W for 60 s using an air plasma was performed on the nanogrooved PDMS and the PDMS ring prior to bonding the ring onto the nanogrooved PDMS to create a PDMS construct with a nanogrooved well of defined dimensions (Figure 1E). To strengthen the bond, the PDMS constructs were placed in an oven at 65 °C for 1 h after plasma oxidation.

For CTX cell culture experiments, patterns with a 600 nm pattern periodicity and 230 nm ridge width were copied directly from the nanoresist mold. The same preparation method of the PDMS constructs were applied for the CTX cell culture experiments as well.

Atomic force microscopy (AFM) was used to verify the presence and fidelity of the nanogrooved patterns in the PDMS constructs. The XE-100 (Park Systems, Santa Clara, CA, USA) with non-contact cantilever tips (PPP-NCHR, Park Systems) was used to assess the nanogrooved patterns used for SH-SY5Y cell culture. An AFM (Bruker, Santa Barbara, CA, USA) with FastScan cantilever tips (Bruker) was used to assess the nanogrooved patterns used for the CTX cell culture.

### 2.2. 3D Cell Culture on Nanogrooved Substrates

#### 2.2.1. 3D SH-SY5Y Cells

After fabrication, the PDMS constructs were placed into the wells of a 24-well plate and subsequently sterilized by submersion in 70% ethanol for 5 min. Next, each construct was washed three times using sterilized, deionized water. The well of the PDMS constructs was coated with 10 µg/cm fibronectin (FC010, Sigma Aldrich, St. Louis, MI, USA) in phosphate buffered saline (PBS; LO BE02-017F, Westburg, Leusden, the Netherlands) for 30 min. Following that, the fibronectin coating was aspirated from the PDMS substrate, and the cell suspension was immediately dispensed on the surface.

The cell suspension consisted of the human neuroblastoma SH-SY5Y cell line (94030304, Sigma Aldrich), a reductionist model of brain cells. Cells were taken from cryovials in liquid nitrogen and thawed, after which the cells were plated in a T75 flask. Cells were kept in growth medium, composed of Dulbecco’s modified Eagle’s medium and Ham’s F12 medium at a 1:1 ratio (L0093-500, VWR, Amsterdam, the Netherlands) supplemented with 10% fetal bovine serum (SFBS lot 11113, Bovogen, East Keilor, Australia) and 1% penicillin/streptomycin (LODE17-602E, Westburg). The cells were kept in an incubator at 37 °C and 5% CO_2_ until the cell culture reached 70–80% confluency. At the start of the experiments with the PDMS constructs, cells were trypsinized, counted and then seeded onto the fibronectin-coated substrates in growth medium at 20,000 cells/cm^2^.

As cells were seeded in a 2D fashion onto the nanogrooved surface of the PDMS constructs, a hydrogel layer covering the cells was subsequently added. For the hydrogel layer, growth factor-reduced Matrigel (734-0269, VWR) was used. Cell cultures were left for at least 3 h, during which cells could adhere to the PDMS cell culture substrate. An hour prior to adding Matrigel to the cell culture, the Matrigel was thawed on ice. When cells had been inspected for substrate adhesion and the Matrigel was fully thawed, the medium was taken off the cell culture and Matrigel was added into the well of the PDMS construct. The cultures where then placed in an incubator at 37 °C and 5% CO_2_ for 15 min for the Matrigel to gel, after which 1 mL of growth medium was added to the wells of the 24-well plate hosting the PDMS constructs.

At 1 days in vitro (DIV), the growth medium was replaced with growth medium supplemented with 10 µM retinoic acid (RA; R2625, Sigma Aldrich), so as to initiate neuronal differentiation [24,25]. The medium supplemented with RA was maintained in the cell cultures for 72 h and refreshed halfway. Subsequently, cell differentiation was further enhanced by adding growth medium with 50 ng/mL brain-derived neurotrophic factor (B2795, Sigma Aldrich) for 72 h [26], with the medium being refreshed halfway. After differentiation, cells were kept in growth medium, with the growth medium being refreshed every other day. At 21 DIV, cell cultures were fixed using 3.7% formaldehyde in PBS for 1 h prior to immunofluorescence staining. During cell culture, cells were observed using an EVOS FL microscope (ThermoFisher Scientific, Eindhoven, The Netherlands).

#### 2.2.2. 3D CTX Cells

The PDMS constructs for culturing CTX cells were coated with a non-protein-based polymer to enhance the cell-substrate adhesion. Branched polyethylenimine (PEI; approximate molecular weight 60,000, 50 wt % aq. solution; Acros Organics, Morris Plains, NJ, USA; CAS: 9002-98-6) was used as the coating material [27]. The PEI coating solution was prepared with a concentration of 50 μg/mL in sterile MilliQ water. The PDMS constructs were first sterilized by submersion in 70% ethanol for 5 min and air-dried, then treated with oxygen plasma and subsequently immersed in the coating solution at 37 °C for a minimum duration of 2.5 h or overnight. Before culturing, the residual coating solution was aspirated in a biological safety cabinet. The PDMS constructs were then air-dried and ready for cell culturing.

CTX cells were isolated from new-born rat’s brain (Mother rat: Wistar Crl:WU) and dissociated in R12H medium [28], then seeded on the coated PDMS constructs with an approximate number of 3 × 10^5^ cells/well (6000 cells/mm^2^). After seeding, the 2D seeded CTX culture was kept in a cell incubator at 37 °C, 5% CO_2_ and 95% humidity for the first 24 h. At 1 DIV, Matrigel was diluted with R12H medium to 75% of its original concentration and subsequently added to the 2D CTX culture, in the same way as it was for the SH-SY5Y cells. Without any differentiation treatment, the CTX cells were cultured with R12H medium containing 100 units penicillin/streptomycin, at 37 °C, 5% CO_2_ and 95% humidity. The medium was refreshed every 2 days until the culture was terminated at 14 DIV. Cell cultures were fixed using 4% formaldehyde solution diluted from 37% formaldehyde aqueous solution (Sigma Aldrich) to 1/8 (v/v) with PBS (Sigma Aldrich, D8537) for 1 h prior to immunofluorescence staining.

### 2.3. Immunofluorescence Staining and Imaging

#### 2.3.1. SH-SY5Y Cells

Immunofluorescence staining was performed on the cell cultures. The SH-SY5Y cells were stained using anti-β-Tubulin III (T8578, Sigma Aldrich) and anti-mouse IgG Alexa 555 (A21424, Molecular Probes, Eugene, OR, USA) as primary and secondary antibodies to selectively stain SH-SY5Y that differentiated into a neuron-like phenotype [29]. Cells were permeabilized for one hour with 0.1% Triton X-100 (Merck Millipore, Burlington, MA, USA), then washed twice for 30 min with PBS. Then, for four hours a blocking buffer of 10% FBS in PBS was applied, followed by incubation overnight with 1:100 primary antibody and 10% FBS in PBS. Cells were washed four times for 2 h with 10% FBS in PBS, then incubated overnight with 1:100 secondary antibody and 10% FBS in PBS. Additionally, 2 drops/mL of NucBlue™ (Thermofisher Scientific) and 2 drops/mL of Actingreen™ (Thermofisher Scientific) were added to the secondary antibody solution to stain the cell nuclei and the cytoskeletal protein F-actin, respectively. Finally, samples were rinsed four times for 1 h with PBS prior to imaging. To ensure cell cultures would not dry out during confocal imaging, PBS was replaced with the embedding medium Mowiol (Sigma Aldrich), which is typically used to mount 2D samples on cover slips prior to imaging, but can also be used for 3D samples. Z-stack images of the stained cells were obtained using a confocal laser scanning microscope (Leica TCS SP5X, Leica, Milton Keynes, UK).

#### 2.3.2. CTX Cells

Fluorescent immunostaining was performed on the CTX cell culture to identify the major cell types within the neuronal networks. Glial fibrillary acidic protein (GFAP) and microtubule-associated protein 2 (MAP2) were marked as specific indications for astrocytes and neurons, respectively. Anti-GFAP antibody (goat; Sigma Aldrich, SAB2500462; 1:200) and anti-MAP2 antibody (mouse; Sigma Aldrich, M9942; 1:200) were used as the primary antibodies. Anti-goat IgG (H + L), CF^TM^ 488A (donkey; Sigma Aldrich, SAB4600032; 1:500), anti-mouse IgG (H + L), both highly cross-adsorbed, and CF^TM^ 640R antibody (donkey; Sigma Aldrich, SAB4600154; 1:500) were used as secondary antibodies. The nuclei of the cortical cells were stained with DAPI (FluoroShield^TM^, ImmunoBioScience Corp., Mukilteo, WA, USA). The stained cell cultures were imaged with a confocal laser scanning fluorescence microscope (Nikon A1 confocal, Nikon, Tokyo, Japan). Imaging of 3D cultures was realized by the z-stage stack scanning in the confocal scanning mode. The images were analyzed with the NIS Elements Analysis software provided by Nikon, and Fiji (Image J) software provided by NIH (Bethesda, MA, USA).

### 2.4. Cell Culture Analysis: CTX Set and SH-SY5Y

**Figure 2:**
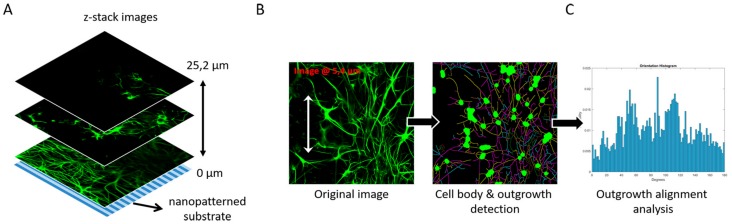
Overview of image-based screening analysis on z-stack images from a 3D neuronal cell culture. (**A**) Schematic example using several image slices from a z-stack showing astrocytes (green) in a primary rat brain CTX culture on top of a nanogrooved substrate. Astrocytes and their outgrowths can be seen across the multiple slices, showing their presence farther away from the nanogrooved surface. (**B**) Cell image analysis software (HCA-Vision, CSIRO) was used to identify cell bodies and their respective outgrowth for each of the slices in the z-stack. (**C**) The identification of outgrowths in (**B**) was analyzed using a Frangi vesselness algorithm to determine the percentage of outgrowths aligned to the underlying nanogrooved substrate for each slice of the z-stack to determine the degree of alignment as function of the distance away from the nanogrooved substrate.

An automated image-based screening method developed by our group [22] was tested toward its use for 3D z-stacks, instead of the originally intended use of 2D cell culture images (Figure 2). In brief, the screening method takes slices at an approximate interval of 1.8 µm from a z-stack (Figure 2A) and uses these images in neuronal cell image analysis software (HCA-Vision, CSIRO, Canberra, Australia) to identify cell bodies and cell outgrowths (Figure 2B). From this automated process, numerous parameters can be extracted, but here the focus lies on determining the number of cells and the length of the outgrowths. The cell bodies are then subtracted from the original image, which results in images with only outgrowths present. A Frangi vesselness algorithm determines the orientation of all of the outgrowths (Figure 2C). Outgrowths are considered aligned when the orientation is <30° relative to the underlying nanogrooved pattern, implying that isotropic orientation of outgrowths is at 33% alignment. Together, the number of cells, outgrowth length and outgrowth alignment describe how the 3D neuronal cell network is shaped by the cells and outgrowths, and how these are influenced by the nanogrooved substrate.

Testing of the image-based screening method was done by first using the method on the data from a 3D CTX culture experiment. Previously, this data was analyzed manually and only generated results with regard to the alignment of the astrocyte outgrowths [7]. Here, the screening method was used on the same dataset to generate results on the outgrowth alignment, as well as the number of cells and the total length of all outgrowths for each of the analyzed slices of the z-stack. Based on the assessment of these results, the screening method was also used to analyze these parameters for 3D SH-SY5Y cell cultures (n = 3).

## 3. Results

### 3.1. Nanogrooved Substrate Fidelity

**Figure 3:**
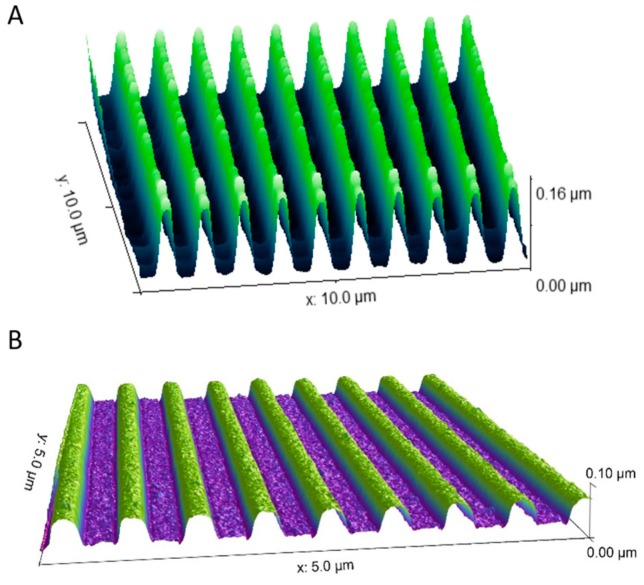
AFM measurements on nanogrooved PDMS substrates. (**A**) Nanogrooved pattern with a ridge width of 246 ± 22 nm and a pattern periodicity of 990 ± 22 nm (n = 5). This pattern was used for SH-SY5Y cell culture experiments. (**B**) Nanogrooved pattern with a ridge width of 219 ± 5 nm and a pattern periodicity of 587 ± 0 nm (n = 5). This pattern was used for primary rat brain cortical cell culture experiments.

The nanogrooved wells of the fabricated PDMS constructs for 3D neuronal cell cultures were measured to assess the nanogrooved pattern fidelity. An AFM measurement was performed for the pattern with a ridge width of 230 nm and pattern periodicity of 1000 nm, as used in the SH-SY5Y cell culture (Figure 3A), resulting in a pattern with an actual ridge width of 246 ± 22 nm and a pattern periodicity of 990 ± 22 nm (n = 5). The same was done for the pattern with a ridge width of 230 nm and a pattern periodicity of 600 nm, as used in the CTX cell culture (Figure 3B), resulting in a pattern with an actual ridge width of 219 ± 5 nm and a pattern periodicity of 587 ± 0 nm (n = 5). Qualitatively, both patterns showed good fidelity with regard to overall pattern dimensions.

### 3.2. 3D SH-SY5Y Cell Culture

**Figure 4:**
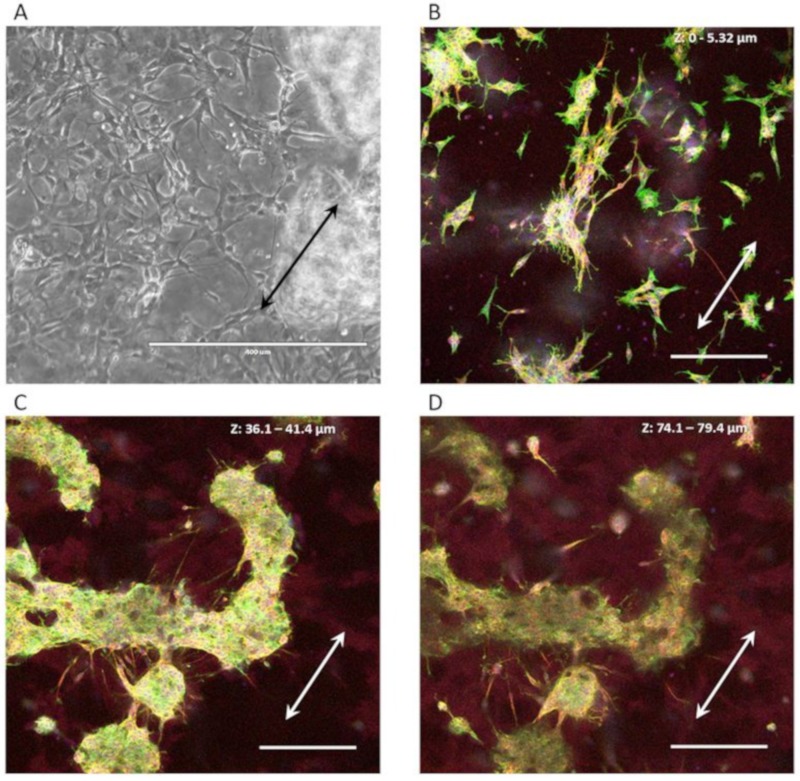
Aligned 3D SH-SY5Y cell cultures on nanogrooved substrates. (**A**) SH-SY5Y cells at 11 days in vitro (DIV) showing differentiation into the neuronal lineage during the experiment, as seen from the neuronal outgrowths. Cells show a qualitative preference to outgrowth direction along the nanogrooves (direction of the double-headed arrows). Blurry areas in the image indicate “clusters” of cells that have migrated into the 3D hydrogel away from their original position on the nanogrooved substrate. The scale bar denotes 400 µm. (**B**–**D**) Maximum intensity projections of subsections of slices from a z-stack showing the extent of SH-SY5Y cells and their outgrowths throughout the 3D cell culture. Qualitatively, cell bodies and outgrowths can be observed to align to the nanogrooved substrate at close range (**B**), with the effect decreasing as the distance to the substrate increases (**C**,**D**). The white, double-sided arrows denote the orientation of the nanogrooves. Scale bars denote 200 µm. Immunofluorescence staining shows neuron-specific staining for β-Tubulin III in red, F-actin in green and cell nuclei in blue.

The SH-SY5Y cell culture experiments were observed for cell survival during the duration of the experiment (Figure 4A). Cells exhibited neuronal differentiation, as seen from the neuronal outgrowths. Also, cells would migrate into the 3D microenvironment, seen from the cells that were out of focus during bright field microscopy of the running experiments when focusing on the cells on the nanogrooved substrate. After immunofluorescence staining, confocal microscopy of the cells (Figure 4B–D) showed that indeed part of the cell population had migrated into the hydrogel, with cells visible up to ~80 µm distance from the nanogrooved substrate. Qualitatively, outgrowths could be observed to preferentially orient along the nanogrooved pattern (Figure 4B) when close to the substrate’s surface. This alignment effect decreased the farther away as cells were from the surface (Figure 4C,D).

### 3.3. 3D CTX Cell Culture

#### 3.3.1. Neuron-Astrocytes Alignment in 2D Culture

**Figure 5:**
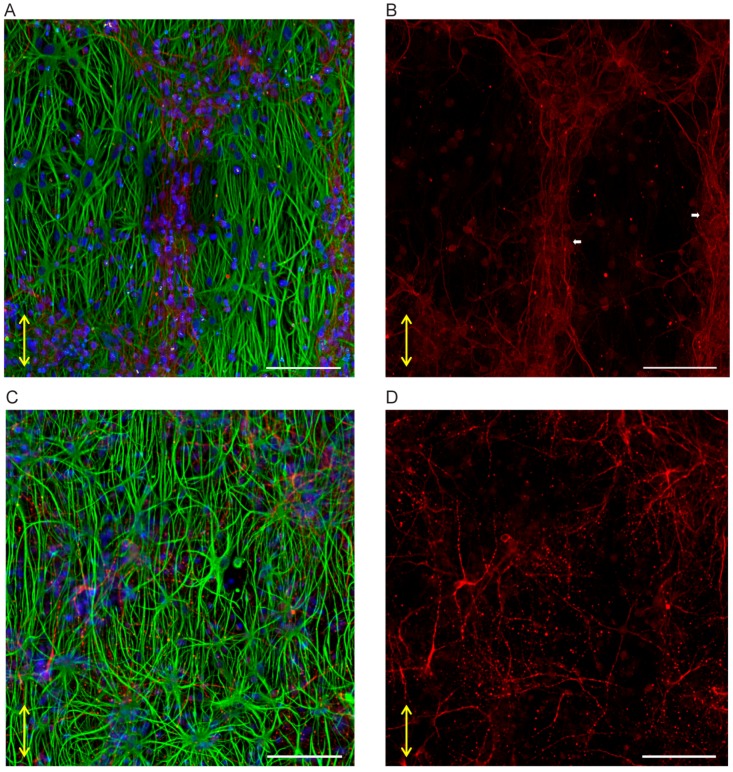
Different neuronal network formation in CTX culture on nanoscaffolds. (**A**,**B**) Outgrowth alignment of the astrocytes and neurite bundles formation on the soft PDMS nanoscaffold. The white arrows in (**B**) indicate the neurite bundles that aligned with the directional outgrowth of the astrocyte. (**C**,**D**) Outgrowth of astrocytes and neurites on the rigid silicon scaffold. Red staining indicates the microtubule-associated protein 2 (MAP2) of neurons, green staining indicates the glial fibrillary acidic protein (GFAP) of astrocytes, and blue staining by DAPI indicates the cell nuclei. Scale bars denote 100 μm. The double-sided yellow arrows indicate the direction of the nanogrooves. Adapted from Figure 4.7 in [30].

Our initial study on the CTX culture has shown associated neuronal outgrowth, also referred to as neurites, with directional growth of the astrocytes on a soft linear nanoscaffold, i.e., PDMS nanogrooves. It has been demonstrated in our previous work that a softer scaffold material such as PDMS seems to affect the growth of the astrocyte and its network formation in a 2D culture [23], owing to the lower stiffness of the PDMS (Young’s modulus 0.01–1 MPa level) than the rigid silicon (Young’s modulus at GPa level). Interestingly, further morphology evaluation on the neurons in the mixed co-culture has shown that the neurons growing on the soft scaffolds have a tendency to form neurites in parallel to the directional outgrowth of the astrocytes., Meanwhile, the neurons on a rigid silicon scaffold do not show similar behavior, despite the outgrowths of the astrocytes showing similar alignment behavior on these two materials (Figure 5A,C). As reported before, there was no distinguishable difference in the astrocyte network formation on the 10:1 PDMS and the silicon [23]. In addition, the organization of the neuronal cell bodies appears to be associated with the “clustering” of the astrocyte’s cell bodies. The cell “clusters” and the neurite bundles interconnect with the adjacent ones, resulting in a neuronal network with a few hundred micrometers of interspacing (Figure 5B), which displays similar organization as the neuron bundles in the brain cortical layers. Again the unique phenomenon was not observed in the culture on silicon scaffolds (Figure 5C,D).

#### 3.3.2. Neuron-Astrocyte Alignment in 3D Culture

**Figure 6:**
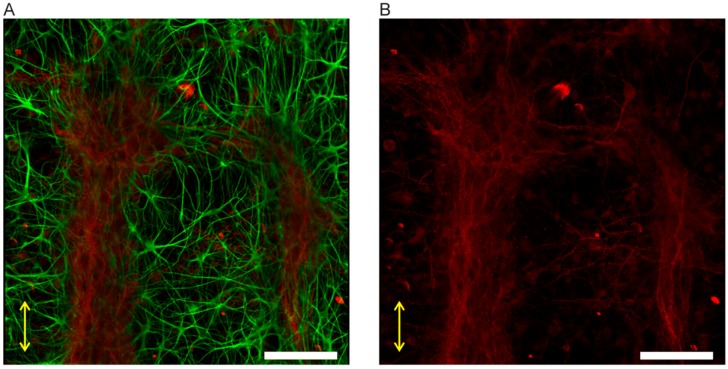
Neurite bundles in 3D-like culture. (**A**) Green staining indicates the GFAP for astrocytes, and red staining (shown alone in (**B**)) indicates the MAP2 for neurons. The double-sided yellow arrows indicate the direction of the nanogrooves. Scale bar: 100 μm. Adapted from Figure 5.11 in [30].

We have previously demonstrated our concept of nanogroove-enhanced hydrogel scaffolds for 3D neuronal cell culture using the combination of nanogrooves and Matrigel to emulate the environment of the extracellular microenvironment, in vivo, applying a manual analysis [7]. As reported in this previous publication, approximately 50% of the outgrowth alignment was still observed in the astrocytes, which had migrated into the Matrigel and were ~6 µm away from the surface of the nanogrooves acting as a scaffold. In comparison, the anisotropic distribution of outgrowths, as seen for the cells on nanogrooved substrates, is not present in controls where the used substrate is flat (Figure S1A), even when cells migrate away from the substrate into the Matrigel (Figure S1B). As for the neurons, the similar aligning behavior of the neurites remained in the 3D culture: the associated neurite bundles in Figure 5 can also be observed in the cells that are growing in the gel. The results suggest that, within the gel scaffold, the aligned neurite bundles are able to maintain ~20 μm height from the bottom into the 3D hydrogel (Figure 6), which corresponds with 2~3 layers of cells. This indicates that the neurite organization formed at the cell-nanogroove interface is stronger than the adhesion between cells and anchoring points in the gel, wherein cells are capable of migrating into the gel space as far as ~80 μm away from the nanogrooved substrate surface (Figure S2A).

### 3.4. Image-Based Screening Method Analysis Using 3D CTX Data Set

**Figure 7:**
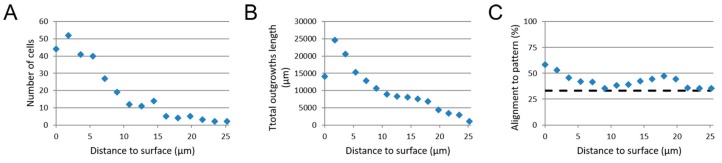
Results from analysis of 3D CTX culture on a nanogrooved substrate. (**A**) The number of cells, (**B**) the total outgrowth length (µm) and (**C**) outgrowth alignment (%) relative to the distance (µm] away from the nanogrooved substrate. The dashed line at 33% alignment in (**C**) represents an isotropic distribution of outgrowths without preferred direction.

As a means to test the image-based screening method against the manually performed measurements published elsewhere already [7], the screening method was used to analyze the 3D CTX data set as a pilot in this work. Quantitative results were obtained for the following parameters: the number of astrocytes, total outgrowth length and the outgrowth alignment relative to their distance from the substrate surface (Figure 7). The number of astrocytes at the nanogrooved substrate was around the 48 cells, whereas after this initial layer of cells near the surface, the number of cells dropped to approximately 13 cells around 10–15 µm from the surface, up to roughly 3 cells at 20 µm and farther away from the surface (Figure 7A). The total outgrowth length is around 14,097 µm at the substrate surface, but then peaks at approximately 24,643 µm, after which there is a sharp decline to 8930 µm around 8 µm and a gradual decline thereafter to 1054 µm at 25.2 µm (Figure 7B). The outgrowth alignment to the underlying nanogrooved pattern is 58.5% at the substrate surface and declines to 35.5% at around 8 µm distance from the surface and a gradual increase to 47.1% at around 18 µm before receding to 35.5% at 25.2 µm (Figure 7C). Compared to the manual analysis previously performed [7], additional information about the cell culture was obtained in the form of the number of cells and the total outgrowth length present in the z-stack. Since the bottom surface of a sample cannot perfectly coincide with the first slice image in a z-stack, the initial values for the number of cells and total outgrowth length are lower compared to the following values found for the next slice image. The decline of all three parameters indicates the decrease of cells when investigating areas farther away from the substrate surface, which coincides with the experimental setup of culturing cells first in a 2D format, prior to adding the hydrogel for the 3D microenvironment.

Considering the lack of detectable outgrowths in particular, z-stacks were not analyzed beyond the 25.2 µm range from the substrate surface upwards. Although cells were detected up to ~80 µm away from the substrate, these were not considered for the investigation of the structural complexity that nanogrooved patterns could induce in the neuronal cell cultures. The results for alignment, shown in Figure 7C, indicate that up to a height of approximately 6–8 µm the screening method also finds outgrowths to be preferentially aligned with the nanogrooved pattern.

### 3.5. Image-Based Screening of 3D SH-SY5Y Cell Cultures

**Figure 8:**
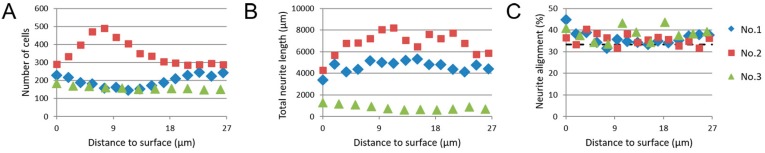
Results from analysis of 3D SH-SY5Y cell cultures on nanogrooved substrates. (**A**) The number of cells, (**B**) the total outgrowth length (µm) and (**C**) outgrowth alignment (%) relative to the distance (µm) away from the nanogrooved substrate. The dashed line at 33% alignment in (**C**) represents an isotropic distribution of outgrowths without preferred direction. For visibility of the data points, the alignment axis was capped at 50% instead of 100% alignment. The legend to the right of (**C**) describes the samples as shown in (**A**–**C**).

Based on the results as shown in Section 3.4, the image-based screening method was considered an appropriate way of generating quantitative data on z-stacks from 3D SH-SY5Y cell cultures on nanogrooved PDMS substrates. Again, results were acquired for the parameters of the number of cells, total outgrowth length and outgrowth alignment relative to their distance from the substrate surface (Figure 8). Three z-stacks were analyzed, numbered 1 through 3.

A range of 151–490 cells can be observed, regardless of distance from the substrate surface for the three z-stacks that were analyzed. For both z-stack 1 and 3, little change was observed in the number of cells relative to the distance to surface. A peak could be observed for z-stack 2 around 7.6 µm, with approximately 190 cells more relative to cell numbers at the surface or farthest from the surface. This is likely caused by the 3D SH-SY5Y cell culture sample being inserted slightly skewed into the confocal microscopy. As a result, cells adhering to the substrate surface seemed to be spread out over several slices within the z-stack when imaged, which most likely has resulted in a higher number of cells being farther away from the surface than anticipated.

As with the number of cells, the total outgrowth length, as seen within the slices of the z-stacks, changes only slightly for z-stacks 1 and 3, averaging at 4636 µm for z-stack 1 and 855 µm for z-stack 3. Z-stack 2 shows a total outgrowth length starting at 4307 µm, increasing to 8214 µm at 11.3 µm distance from the surface, before declining to 5871 µm at 26.4 µm away from the surface. These values can be compared against our previous experiments, which were on 2D SH-SY5Y cell cultures on nanogrooved PDMS substrates, and performed with a similar experimental protocol, but without the addition of the Matrigel to create 3D cell cultures [22]. In those experiments, we considered parameters such as the percentage of differentiated cells and the average outgrowth length of differentiated cells, as opposed to the total number of cells and the total outgrowth length. When taking the values for the total number of cells and the total outgrowth length from the previous dataset (Table S1), which have not been assessed in a context such as here, the number of cells and the total outgrowth length within the 3D SH-SY5Y cell culture are comparable to the 2D SH-SY5Y cell cultures.

The outgrowth alignment to the nanogrooved pattern was, for all z-stacks, in the range of 33.5–44.8%, with a higher percentage of alignment mostly occurring up to 11.3 µm away from the surface. While comparatively low in alignment percentage, as compared to the results of the CTX cells, the alignment percentages do show that some preference for outgrowth direction along the nanogrooves is retained for these reductionist type of neuronal cell cultures.

## 4. Discussion

In this work, we have aimed to demonstrate that it is possible to control outgrowth direction in 3D by simply applying soft scaffolds, such as Matrigel, atop of neuronal cell cultures initially seeded on nanogrooved substrates. In the case of the reductionist human neuronal cell line SH-SY5Y, results show that a layer of cells cultured in a 2D conformation will migrate into their 3D environment and also retain a degree of alignment to the underlying nanogrooved PDMS substrate. To our knowledge, results for neuronal cell cultures in 2D find similar topographical guidance effects of nano- and micro-scale features on neuronal cell outgrowths and morphology [14,31,32,33], however these do not investigate the emergence of structural complexity in 3D. The approach of a reductionist cell model allows for a more straightforward investigation of the neuronal cell morphology and neuronal network formation as opposed to the complex nature of primary cell cultures or co-cultures. As seen from the confocal imaging z-stack shown in Figure 4, cells can form dense cell clusters, and background interference from the hydrogel’s autofluorescence can interfere with the quantitative analysis of such images. The cell clusters are even more challenging for assessment when dealing with multiple cell types, such as the CTX cultures. In this particular case, the autofluorescence of the hydrogel is due to the formaldehyde fixation of the Matrigel. Recent work into clearance techniques to enhance visualization of 3D organoids [34,35] may prove beneficial toward better visualization, thereby alleviating these challenges. In turn, improved visualization may lead to more capabilities in the quantitative analysis of neuronal cell culture images, such as investigating the branching of outgrowths, neuronal polarity and for co-cultures the number of cells per cell type, e.g., neurons, astrocytes. The current image-based screening method works by analyzing the slices from each z-stack separately. Typically, the resolution of data across slices in the z-direction is limited and relatively noisy compared to the data within each slice in the x- and y-direction. This effect currently limits both the usefulness and implementation of whole 3D analysis algorithms.

Compared to the CTX cell culture, the SH-SY5Y cell line provides a reductionist human brain model, but without the interplay complexity of co-cultured glia cells and neurons. This reduction allows for a thorough analysis of neuronal network formation parameters dependent on differentiation-stimuli at the cellular scale. Although we have used different dimensions of the nanogrooved patterns for CTX cells and SH-SY5Y cell culture experiments [21,22,23,24,25,26,27,28,29,30], these different patterns elicited similarly high responses with regard to outgrowth alignment, which was essential for analyzing the feasibility of our approach. Also, this shows that the application of a reductionist neuronal cell model, here the SH-SY5Y cell line, can still express structural complexity in nanogroove-enhanced hydrogel scaffolds. On the other hand, these simple cell line models do not show the emergence of “meta-structures” in 3D conformation by applying our 3D culture format. Although neurite bundles, as seen in Figure 5 and Figure 6, did not emerge from the SH-SY5Y cells in our experiments, such reductionist human brain models still open up possibilities to investigate the changes in neuronal network behavior, due to pharmacological interventions and set-up experiments at a scale of relevance for the industry, with a predefined set of clear parameters. In particular, our quantitative findings that compare the results for the number of cells and the total outgrowth length between 2D and 3D SH-SY5Y cell culture, as described in Table S1, can be used for setting up a baseline for optimization procedures toward robust system design and to establish a baseline expectation from cell culture results.

With regard to the CTX cultures, it has been reported that astrocytes can act as a layer of structural “scaffold” for directional neurite growth on 2D surface [36,37], where the environment lacks biochemical cues for neuron adhesion. In those peer studies, the neuron-astrocyte co-culture was realized by seeding the astrocytes first, followed by the neurons on top, separately. Instead of directly contacting the culturing substrate, the neurons face the “natural” surface of the pre-allocated astrocytes, and aligned neurites were thus induced by the morphology of the astrocytes. A similar phenomenon was observed in our study as well. In Figure S3, immunostaining of GFAP and MAP2 in the gel indicates the interconnection between astrocyte outgrowths and neurites. The neurite grows along with astrocyte outgrowth, extended in parallel direction within the gel in different layers of height. The step of the z-stage stack scanning was 300 nm, which is thinner than the average diameter of these branches [38]. The parallel trajectory of neurites and astrocyte outgrowths suggests that the astrocyte outgrowth may still assist or regulate the neuritogenesis or neurite orientation [39,40,41,42,43] with the presence of the extracellular environment provided by the Matrigel.

The unique associated network formation observed in our experiments using a combination of hard and soft scaffolds suggests the possibility of realizing brain-like architecture in vitro. This happens through the assistance of the directional guidance from the culturing environment: The hard nanogrooved “scaffold” at the bottom of the culture provides topographical information of the directional growth for the cells in a strongly determined manner, however remains effective in guiding the neuronal network formation in a few layers of the cells in the soft gel “scaffold”. This phenomenon is visible for both nanogrooved pattern types, as well as both neuronal cell cultures. It suggests that the nanogrooves provide mechanical boundary conditions that limit the degree of freedom by strong cell–cell interactions also in the gel. The gel layer, on the other hand, offers spatially distributed anchor points for the cells in 3D with additional biochemical cues, facilitating the formation of an extended neuronal network throughout the culture volume. The bottom layer of cells in the culture, contacting the nanogrooved substrate, plays a crucial role as an interface connecting the two types of scaffolds.

A potential limitation of this 3D construction, regardless of the chosen neuronal cell model, would be the lack of sufficient circulation of the nutrition supply and the waste clearance during the culturing. In the experiments discussed in this work, the culture medium was only refreshed from the top of the Matrigel layer. Although the Matrigel allows the diffusion of the nutrients efficiently, the physical confinement of the gel layer may hinder the removal of the debris of the dead cells during the refreshment of the medium. It would, at the least, be less efficient than the same process in the conventional 2D cultures. This may result in accumulation of the waste biochemicals, particularly on the bottom of the culture, where the majority of the cells are located, as has been observed for primary rat CTX cells (Figure S2B). Conversely, the data shown in Table S1 suggests that the volume of Matrigel used for the 3D SH-SY5Y cell cultures was not detrimental with regard to nutrition supply, as the overall number of cells or outgrowths were within a similar range compared to 2D SH-SY5Y cell cultures. Nevertheless, an integrated microfluidic culturing chamber, additional to the 3D constructions, may provide improvement by creating a robust platform for the circulation of medium in the culture environment, and therefore may result in a higher consistency of the cell viability [44,45].

Furthermore, to seed a cell suspension within the gel onto the 2D cell layer could achieve an even more realistic 3D culture, also then offering multiple cell types. Alternatively, to prepare 3D scaffolds with nano- to micro-scale topographical guidance cues such as fiber-based scaffolds and microtunnels [46] can also help to realize a more advanced 3D culturing model. Other approaches have also been used to realize a more brain-like 3D neural cell culture, such as mixing neuronal cells in hydrogel and seeding the 3D construct on a petri-dish in a conventional way [47], as well as bioprinting neuronal cells or tissue within a medium or hydrogel [48,49]. Also, the layered 3D construction in cerebral cortex can be realized by stacking layers of cells confined in gel [50], however, the reproduction of the “cortical column” has not yet been discussed. Pluripotent stem cells are a promising cell type for realizing brain organoids, which have shown ability to reproduce both structural and functional properties of the brain cortex [51], while the formation of distinct cortical neuronal layers, as well as complex neuronal circuitry, still remain a challenge [52]. Despite these alternatives, nanogrooves can provide a valuable alternative from a system’s design point of view, where the platform itself has integrated features, here nanogrooved patterns, to add structural complexity to 3D neuronal cell cultures. Such purely passive features, which can be an integral part of a standard culture wells plate or microelectrode array, are especially useful for high-throughput biological assays in a commercial setting. As stated in the previous paragraph, the use of an integrated microfluidic culturing chamber could extend the capabilities of such brain models, not only by providing enhanced nutrient and waste circulation, but also through the possibility of implementing multiple nano- and micro-scale features to enhance both formation of the brain cell model and the functional readout thereof. In turn, our results aid these models to potentially achieve such a level of control over the brain cell culture that specific brain regions, or structures such as cortical columns, can be mimicked in vitro.

## 5. Conclusions

In this work, we developed a method of adding structural network complexity in 3D in vitro neuronal cell models. This was performed by culturing neuronal cells on nanogrooved substrates covered with Matrigel. Two neuronal cell models, primary rat brain cortical cells and SH-SY5Y cells, were analyzed using image-based screening, for which the quantified results show that cells create a network in the 3D microenvironment and retain a degree of approximately 38–58% alignment, with regard to the underlying nanogrooved substrate in the z-direction for several micrometers. Also, the indication that 3D neuronal cell cultures that maintain a range of approximately 150–350 cells per mm^2^ with an approximate total length of 4500–7500 µm of outgrowths, allows for accessible parameters to investigate during further optimization of a robust platform. In conclusion, these results indicate that nanogrooves enhance the structural complexity of 3D neuronal cell cultures for both primary rat brain cortical cell cultures and human SH-SY5Y cultures, providing a basis for advances in brain-on-chip technology.

## Supplementary Materials

The following are available online at https://www.mdpi.com/2072-666X/10/10/638/s1, Table S1: Parameter values derived from 2D and 3D SH-SY5Y cell cultures on nanogrooved PDMS substrates, Figure S1: Neurite and astrocyte outgrowth of the 3D CTX cell culture on a flat PDMS substrate as control, Figure S2: Cell migration and viability in gel in the 3D CTX cultures, Figure S3: Parallel growth of neurite and astrocyte outgrowth in gel.
