# Supplementary material for: Nanogroove-Enhanced Hydrogel Scaffolds for 3D Neuronal Cell Culture: An Easy Access Brain-on-Chip Model
Source: Micromachines (Basel). 2019 Sep 23;10(10):638. doi: 10.3390/mi10100638 (PMC6843116; doi:10.3390/mi10100638)
Supplement: Supplementary file 1 [file micromachines-10-00638-s001.pdf]

micromachines

MDPI

Supplementary Materials

Nanogroove-Enhanced Hydrogel Scaffolds for 3D Neuronal Cell Culture: An Easy Access Brain-on-Chip Model

Alex J. Bastiaens, Sijia Xie and Regina Luttge

Table S1. Parameter values derived from 2D and 3D SH-SY5Y cell cultures on nanogrooved PDMS substrates.

Sample No.	Number of Cells	Total Neurite Length (μm)	Est. Number of Cells in 1 mm^2 [1]	Est. Total Outgrowth Length (μm) in 1 mm^2 [1]
I: 2D SH-SY5Y cell culture on nanogrooves [22]				
image surface area: 0.91 mm^2				
1	227	4083.2	249	4487.0
2	138	4017.2	152	4414.5
3	289	4297.9	318	4723.0
4	299	10852.7	329	11926.1
5	264	6744.3	290	7411.3
II: 3D SH-SY5Y cell culture on nanogrooves				
image surface area: 0.60 mm^2				
1	197 [2]	4636.5 [2]	216	5095.1
2	352 [2]	6766.2 [2]	386	7435.4
3	159 [2]	855.5 [2]	175	940.1

[1] For comparison between images of category I and II where image surface area was taken into account, the values for the number of cells and total outgrowth length were divided by the image surface area corresponding the appropriate category.

[2] The mean values were taken for the number of cells and the total outgrowth length for the analyzed slices of each z-stack sample.

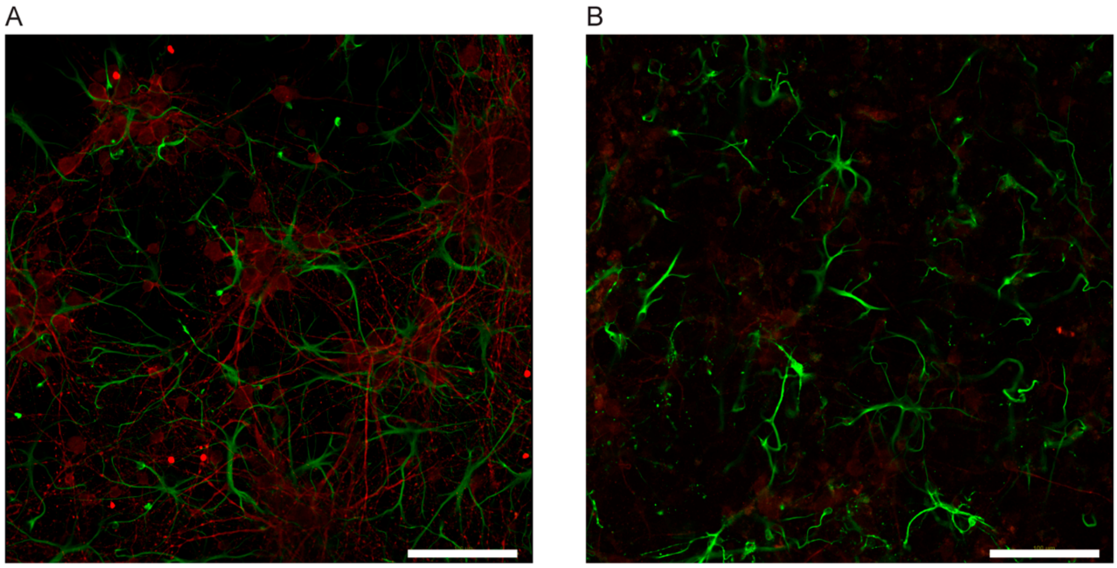

Figure S1. Neurite and astrocyte outgrowth of the 3D CTX cell culture on a flat PDMS substrate as control. (**A**) Cells on the bottom of the culture; (**B**) Cells in gel. Red: MAP2; green: GFAP. Scale bar: 100 μm.

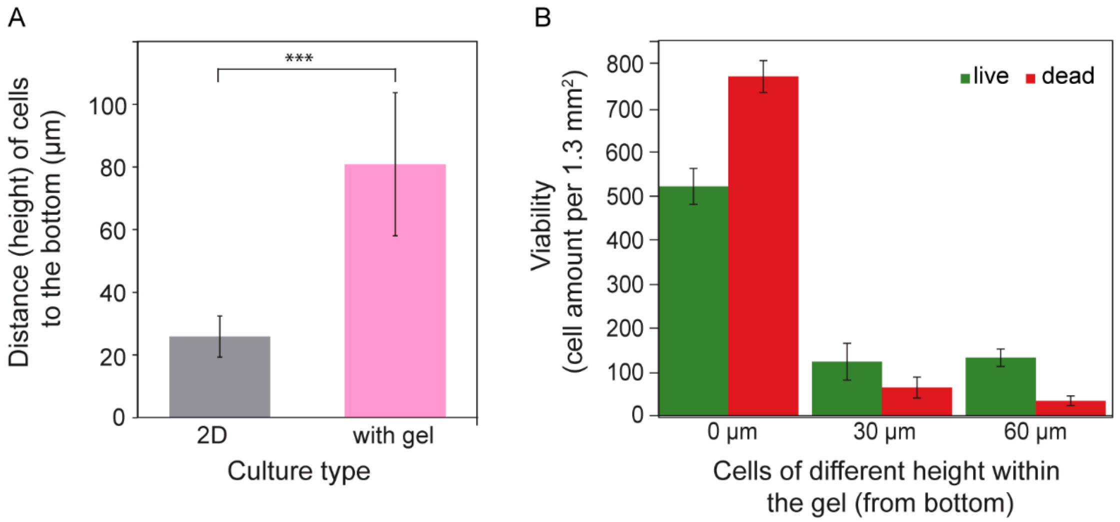

Figure S2. Cell migration and viability in gel in the 3D CTX cultures. (**A**) Comparison of the height position of the DAPI stained single cell nuclei in 2D and 3D CTX cultures, n = 15 (n: individual imaging areas), $p < 0.001$. (**B**) Cell viability at different height positions in the gel at 14 DIV, n = 4.

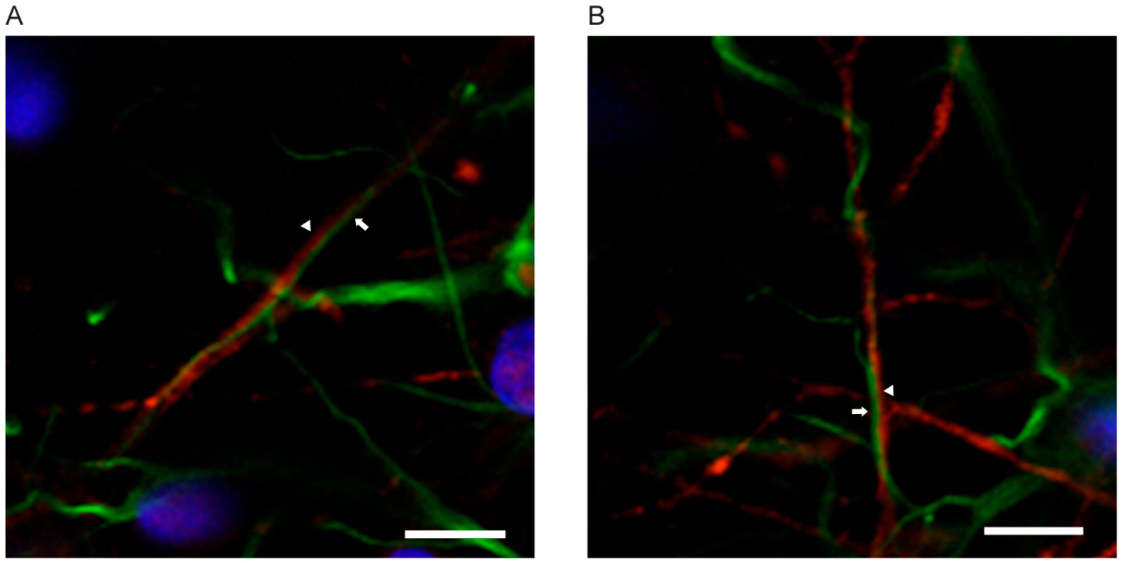

Figure S3. Two images (**A**,**B**) showing parallel growth of neurite and astrocyte outgrowth in gel. The arrow indicates the parallel growth. Red: MAP2 (triangles); green: GFAP (arrows); blue: DAPI. Scale bar: 20 µm.

© 2019 by the authors. Submitted for possible open access publication under the terms and conditions of the Creative Commons Attribution (CC BY) license (http://creativecommons.org/licenses/by/4.0/).
